# SABE Colombia: Survey on Health, Well-Being, and Aging in Colombia—Study Design and Protocol

**DOI:** 10.1155/2016/7910205

**Published:** 2016-11-13

**Authors:** Fernando Gomez, Jairo Corchuelo, Carmen-Lucia Curcio, Maria-Teresa Calzada, Fabian Mendez

**Affiliations:** ^1^Research Group on Geriatrics and Gerontology, International Association of Gerontology and Geriatrics Collaborative Center, University of Caldas, Manizales, Colombia; ^2^Escuela de Salud Pública, Universidad del Valle, Research Group of Epidemiology and Population Health (GEPH), Cali, Colombia

## Abstract

*Objective*. To describe the design of the SABE Colombia study. The major health study of the old people in Latin America and the Caribbean (LAC) is the Survey on Health, Well-Being, and Aging in LAC, SABE (from initials in Spanish: SAlud, Bienestar & Envejecimiento).* Methods*. The SABE Colombia is a population-based cross-sectional study on health, aging, and well-being of elderly individuals aged at least 60 years focusing attention on social determinants of health inequities. Methods and design were similar to original LAC SABE. The total sample size of the study at the urban and rural research sites (244 municipalities) was 23.694 elderly Colombians representative of the total population. The study had three components: (1) a questionnaire covering active aging determinants including anthropometry, blood pressure measurement, physical function, and biochemical and hematological measures; (2) a subsample survey among family caregivers; (3) a qualitative study with gender and cultural perspectives of quality of life to understand different dimensions of people meanings.* Conclusions.* The SABE Colombia is a comprehensive, multidisciplinary study of the elderly with respect to active aging determinants. The results of this study are intended to inform public policies aimed at tackling health inequalities for the aging society in Colombia.

## 1. Introduction

The major health study of the old people in Latin America and the Caribbean (LAC) is the SABE study Survey on Health, Well-Being, and Aging in Latin America and the Caribbean; SABE (from initials in Spanish: SAlud, Bienestar & Envejecimiento) is a multicenter project originally conducted by the Pan-American Health Organization (PAHO) [1–4]. The study included 10,891 individuals, 60+ years of age, living in seven big cities of the region (Bridgetown, Buenos Aires, Havana, Mexico City, Montevideo, Santiago, and Sao Paulo) and was based on a probabilistic, stratified, multistage, cluster-sampling design of noninstitutionalized elderly population of the seven participating cities. The study represented a milestone in the field of population aging in the region and could provide enough information to study the phenomenon of aging in detail. Results of SABE increased our understanding of the aging processes and the formulation of public policy toward the well-being of the LAC populations and provided a solid base for a second generation of studies in the region [2]. For example, on the basis of this research second panel data was analyzed in Chile and Brazil [5, 6].

After the SABE study was conducted in Latin America, several cross-sectional studies have been carried out in the region. Between 2009 and 2010 in Ecuador, a similar study with emphasis on aborigine population (10.4% of total population) was conducted: SABE Ecuador [7, 8]. As expected, ethnicity is a critical factor in poverty, inequity, and social exclusion among aborigines in the region [9]. Recently, with a similar methodology of the SABE study, a cross-sectional survey was conducted in the urban zone of Bogotá (Colombia) including 2000 people of 60+ years [10]. In Peru a SABE study is ongoing by 2016.

More than 40 papers from SABE study have been published during the last decade including different topics like gender, chronic conditions, hypertension, diabetes mellitus, obesity, anemia, cancer, anthropometric measures, oral health, mobility, frailty and sarcopenia, disability, falls, depression, cognitive function, and caregivers [11, 12].

Colombia is a country of approximately 48 million inhabitants, with some 5.2 million being aged 60 years and above. Currently, life expectancy in Colombia is 72.3 years and by 2025 it will be 77.6 years for women and 69.8 years for men. Colombia is experiencing demographic changes including population aging, decreasing fertility, rapid urbanization, and changes in the epidemiological profile with the persistence of communicable diseases and a concomitant increase in noncommunicable chronic diseases [13]. Despite long-term adverse social conditions related to inequities and violence, elderly population has characteristics in their aging process similar to other areas around the world [13]. However, many aspects included in SABE Latin America have not been explored in Colombian older people. The aim of this paper is to describe the design of the SABE Colombia study.

## 2. Materials and Methods

This is a cross-sectional study of community-dwelling elderly individuals living in urban and rural areas of Colombia. The target population for SABE Colombia includes all adults aged 60 years and above who reside in households. Following conventional practice for population surveys, institutionalized persons (of prisons, jails, nursing homes, and long-term or dependent care facilities) were excluded. The methods and procedures were based on those used in the SABE international study to reach comparability [4] but included an analytical approach based on active aging as recommended by WHO to understand factors, processes, or “determinants” that influence individuals, families, and nations over the life course [14].

Individuals for the SABE Colombia were selected following a multistage area probability sampling design. In particular, sampling was developed using a multistage cluster-sampling technique with stratification of the units at the highest levels of aggregation. The sample included four distinct selection stages. The primary stage of sampling was carried out with a probability proportionate to size (PPS) selection of municipalities as primary sampling units (PSUs). The secondary sampling units (SSU) were area segments (i.e., blocks) randomly selected within PSUs. The third stage of sample selection was preceded by a complete listing (enumeration) of all housing units (HUs) that were physically located within the bounds of the selected SSU. The third sampling stage was a systematic selection of housing units from the HU listings for the sampled SSU. The fourth and final stage in the multistage design was the selection of the household unit within a sample HU. The SABE Colombia is integrated within the general framework of the Colombian National Surveys System, which has a common sampling design inside the national master sample (“muestra maestra”) for country population surveys in Colombia. This is the first study of aging in Colombia that is conducted nationwide with a sample framework included in the national survey system. Given the political situation, relevant aspects of violence, displacement, housing, and income networks integrated into a conceptual model from the social determinants of health and active aging were included in the survey.

The estimated sample size was 24553 individuals, and assuming an 80% response the target sample was 30691 individuals. However, at fieldwork after implementing several strategies to achieve the overall sample and prevent nonparticipation, response proportion was about 70% and varied by region and urban/rural distributions. Specifically, data collection took place between April and September 2015, and response proportion ranged from about 62% in urban areas to 77% in rural sites. It should be kept in mind that all interviews were face to face, and the fieldwork included large metropolitan areas, where traditionally there is more reluctance to provide a survey interview than in more rural areas. Also, a very few areas in our country were considered unsafe for fieldwork and were excluded from the sample. In consequence, we are confident that the nonresponse was not related to main issues analyzed in the survey and that the sample obtained represents well all groups and regions of the target population. The final sample size achieved, including 244 municipalities across all departments (i.e., states) of the country, was 23.694 elderly Colombians.

Participants were included if they were 60+ years of age, were capable of communicating with the research team, and provided written informed consent. Individuals were excluded at the beginning of the interview if they had a total score of less than 13 in the revised version of the Folstein Mini-Mental State Examination, known as MMSE [15]. Low scores in the MMSE were considered indicative of inability to complete the study procedures, and therefore a proxy interview was developed. The percentage of interviews applied to proxies was 17,5%.

Active aging is defined as the dynamic, lifelong interplay of risk and protection within the person and within the environment [16]. Active aging has proven to be influential in guiding policies and research agendas throughout the world. In agreement with the original active aging policy framework, we adopted an analytical approach to assess determinants as recommended by the WHO [14]. The approach not only helped us to define operative categories of analysis but also added comparability to our data with research on determinants of active aging produced in North America and Europe.

In particular, for data analysis we grouped variables based on categories of active aging determinants as follows: economic, social environment, physical environment, personal factors, behavioral, and health and social services systems. In addition, given that active aging includes the role of culture and gender as cross-cutting factors influencing all other determinants in the SABE study, we included key analysis variables related with demographic indicators, socioeconomic status, geographical regions, and race/ethnicity.

All data were collected and managed using a database program specifically designed for the SABE Colombia (Synkron, folder synchronization). Quality control and quality assurance procedures were implemented in the field and using telephone verification of registered data to control for erroneous or missing values. Only trained and authorized personnel were able to access the database system. All participants were interviewed and their responses registered on mobile capture devices (tablets), while in some circumstances (insecurity and no internet access to upload questionnaires) printed versions of the questionnaire were applied and later were digitalized. All information was stored using a security protocol to assure safety and confidentiality of collected data. All collected source data are maintained and stored at the Epidemiological Office in National Health Ministry, Bogotá Colombia. Contact information can be found at the following website: https://www.minsalud.gov.co/. The SABE Colombia data set includes a range of sensitive personal information about individuals. It is essential that privacy is protected and that confidentiality is maintained; we use a series of measures to ensure this. Maintenance and use of the data set are overseen by a Steering Committee and every project is considered by this committee. Researchers interested in collaborative work are invited to contact delegate from the Ministry of Health: Herney Rengifo. All information related with results of SABE Colombia is available at https://www.minsalud.gov.co/sites/rid/Lists/BibliotecaDigital/RIDE/VS/ED/GCFI/Socializacion-Resultados-SABE-2016.zip.

Trained health investigators performed physical examination and medical laboratory technicians collected blood samples and laboratory tests. They all were instructed to follow the standardized clinical and laboratory protocols.

Data analyses were carried out using statistical methods appropriated for multistage sampling techniques. Descriptive and inferential statistics were developed taking into account the probability of selection of individuals in the sample to calculate the expansion factors. A focus on social determinants of health is a core component of the data analysis, in order to identify and characterize health inequities among elderly individuals. In particular, the prevalence of various geriatric and chronic conditions and related factors was estimated according to age, gender, ethnicity, place of living, employment, and other key socioeconomic variables to illustrate health disparities among individuals. Statistical analyses were conducted using STATA version 13 (Stata Corp LP, College Station, Texas, USA) and SPSS version 22.0 statistical package (SPSS Inc., Chicago, Ill., USA).

Ethics committees of both University of Caldas and University of Valle reviewed and approved the study protocol. Participants provided written informed consent (including permission to use secondary data and blood samples) before enrolling in the study and completing the first examination. Participants were informed that they could choose to withdraw from the study at any time. Interviews were conducted in a separate room to protect the participants' privacy. The procedures were in accordance with institutional guidelines and were approved by an institutional review committee. Institutional review boards at both universities involved in developing the SABE Colombia approved the study protocol, and a written informed consent was obtained from every individual before inclusion.

## 3. Results

Before starting fieldwork, a pilot study with 84 individuals was conducted to evaluate questionnaire application among different ethnic groups and cultures participating in the study. Standard Operating Procedures (SOPs) were written for all procedures in the protocol and a study manual was set to guide fieldwork. In addition, research and field staff was trained in all aspects of data collection, questionnaire administration, and development of physical measurements. Interviewers received the same standard training based on protocol instructions and data entry forms. In particular, the core research staff coordinated the development of training of all interviewers focusing on the study manual of procedures and using role-playing.

In addition to the questionnaire covering active aging determinants the survey included anthropometric measurements, taking blood pressure, assessment of physical function, and taking blood samples for biochemical and hematological measures. Furthermore, the SABE study was composed also of two additional components (Table 1): (1) a subsample survey among family caregivers and (2) a qualitative study with gender and cultural perspectives of quality of life to understand different dimensions of people meanings.

Table 2 shows the sociodemographic indicators of participants at recruitment in 2015 by residence and by sex.

Data collection and analysis were guided with the following categories of determinants.

### 3.1. Economic Determinants

Socioeconomic factors were assessed with regard to work history: current occupation, most long-term occupation, and reasons for no work at the moment of interview; income: detailed data on subjective and objective financial status, household income, and expenditures; and social protection: pensions and social transfers. Furthermore, marital status and ethnicity were obtained [17].

### 3.2. Social Environment Determinants

Information in this section included educational attainment and degree of literacy; assessment of frequency, size, and closeness of social support and social networks to provide information about the social environment of Colombian older people; living arrangements, social environments, and perception to neighborhood safety. Also, under a life-course perspective, early-life circumstances (first 15 years) and childhood adversity, abuse, mistreatment, and perceived discrimination were assessed to identify disparities across socioeconomic status originated in childhood [18]. Participants were also asked about their social participation in 18 most common activities reported in previous studies, including hobbies and traveling [19]. Because of specific characteristics of violence in Colombian urban and rural regions, emphasis on characteristics of abuse, mistreatment, and social exclusion (perceived discrimination) and reasons of migration and displacement in the last 5 years were also assessed.

### 3.3. Physical Environment Determinants

Enabling and supportive environments for older people were one of the central goals at the Second World Assembly on Ageing, Madrid, 2002. Housing conditions were assessed in a similar way of original SABE study [3]. Items included housing characteristics, housing safety, and environmental risks. Use of technology was explored in relation to access to information and communication technologies and daily use of technological tools such as microwave oven, automatic cashier, or cell phone. Transportation services, built environment, and public services were also assessed.

### 3.4. Personal Determinants

In original active aging determinants, cognitive capacity is included among personal determinants; however, SABE survey included cognitive impairment; as a consequence, it is included in health determinants section. Other determinants as spirituality, sexuality, subjective health status, life space assessment, and functioning were assessed. Functioning was assessed with activities of daily living (ADL) evaluation using two types of questionnaires: a Spanish-adapted version of physical level ADL (Barthel Index) [20] and an adapted instrumental level ADL scale recommended for epidemiological studies in the elderly people [21]. Mobility disability was defined as having difficulty in walking 400 m or climbing a flight of stairs without resting [22]. Grip strength was assessed by using the average of two Takey hydraulic dynamometer (the Smedley Hand Dynamometer III) attempts, and the reported stronger hand category measure was included for analyses. Physical performance was assessed by the validated Spanish version of short physical performance battery (SPPB) [23]. The SPPB includes three timed tests of lower body function: a hierarchical test of standing balance, a 4 m walk, and five repeated chair stands.

### 3.5. Behavioral Determinants

Habits as cigarette smoking and alcohol consumption (current and past behavior) were assessed; current physical activity was assessed by an adaptation of Reuben's Advanced Activities of Daily Living scale [24]. The participants were classified into four categories according to their answers: frequent vigorous exercisers, frequent long walkers, frequent short walkers, and persons who did not exercise frequently (sedentary group).

Nutritional status was assessed by using the longer, original version of the Mini-Nutritional Assessment (MNA) [25]. Anthropometry measurements include height and body weight; a portable stadiometer (SECA 213) and an electronic scale (Kendall platform scale graduated) were used. Body mass index (kg/m^2^) was calculated. Blood pressure and pulse rate were measured using an electronic manometer (HEM-7113, Omron Healthcare Co., Ltd., Kyoto, Japan). Values were recorded after 5 min of rest in the sitting position and three consecutive measures were obtained, waiting for at least 30 s between readings. Waist circumference was measured over the midpoint between the lower border of the ribs and iliac crest in the midaxillary plane. Calf and mid-arm circumferences were measured at the largest point of the calf and arm, respectively. Oral health determinant was assessed by several items including self-reporting of presence or missing of teeth and access to dental care. In the SABE, the Geriatric Oral Health Assessment Index (GOHAI) scale was used to quantify the “unmet needs for oral health services” of older adults [26]. Preventive breast and cervical cancer screening among women and prostate cancer screening among men in the last two years were assessed.

### 3.6. Health and Social Services Systems

Medical information includes multimorbidity, chronic conditions adapted from the original SABE study (hypertension, osteoarthritis, diabetes, cardiovascular disease, osteoporosis, chronic obstructive pulmonary disease, cancer, and stroke), sensory impairments (vision and hearing loss), diseases linked to the aging processes (depression, dementia, falls and fear of falling, urinary incontinence, and frailty), mental well-being, and medications use (polypharmacy, hypnotics, homeopathic products, and adherence). Medication use and access and utilization of healthcare services (hospitalization, out-patients services, and preventive programs use) were evaluated using questions of the Economic Commission for Latin America and the Caribbean (ECLAC) questions set recommended for epidemiological studies in the elderly people [21].

### 3.7. Blood Analysis

After an overnight fast, blood and urine samples were collected in the morning. Blood samples were centrifuged for 10 min at 3000 rpm 30 min after sampling. All samples were delivered to a single central laboratory (Dinamica Laboratories, Bogotá, Colombia) for analysis within 24 h. Biochemical tests include hemoglobin and hematocrit, total, LDL, and HDL cholesterol, and triglycerides. Residual samples were stored at −80°C for future analysis.

### 3.8. Family Caregiving

A family caregiver was defined as a member, relative, or friend at the age of 18 years and above who provides unpaid assistance to an elderly care recipient in at least one ADL or IADL. The caregiver could be staying in the same household or another household (e.g., married daughter). The effects of being a family caregiver, though sometimes positive, are generally negative, with high rates of burden and psychological morbidity as well as social isolation, physical ill-health, and financial hardship. We assessed characteristics of care, perceived burden, caregiver health, and formal or informal training to care for the elderly [27].

### 3.9. Qualitative Data

WHO defines quality of life as individuals' perception of their position in life in the context of the culture and value systems in which they live and in relation to their personal goals, expectations, standards, and concerns [28]. As part of the SABE Colombia, a qualitative study from gender and culture perspectives was developed to understand the quality of life meanings and to evaluate their particular dimensions (interactions and participation, physical and psychological well-being, experiences, relationships, social contacts and support, and living environment). A pilot study was carried out to adapt the procedures and to build a guide for individual interviews and focus groups. Using a symbolic interactionism approach, a total of 123 interviews and 11 focus groups, including men and women, were done in urban and rural areas of Colombia. Data analyses were conducted using ATLAS Ti (for Windows).

## 4. Discussion

This study protocol describes the design and methods used in the SABE Colombia study. The SABE Colombia study is designed to assess socioeconomic variables, health status, nutrition, cognition, depression, anthropometric and biological measures, and social support and networks in community-dwelling elderly persons in Colombia. This broad investigative scope has allowed the sample to be well characterized, collecting an extensive array of factors (biomedical, physical, lifestyle, behavioral, and sociodemographic) that contribute to the health and well-being status of participants. Furthermore, in this study, we focus on not only quantitative approach of epidemiological studies, but also a qualitative approach to identify participant's meanings of quality of life. In addition, blood-sampling approach provided a nationally representative sample and will allow inferences about metabolic and hematological status of Colombian older population. Moreover, samples from participants have also been stored for future analysis.

There are several strengths in this study. First, this study has a large sample size, with enough statistical power to detect mild to moderate associations. Second, this study was well designed with stratified randomized sampling methods, taking place in all geographical areas of Colombia. Third, the use of variables included in active aging determinants permits us to change from “needs-based” approach to a “rights-based” approach of older people; we need a better understanding of the pathways that explain how these broad determinants actually affect health and well-being in developing world. Fourth, the face-to-face interview method may be useful in obtaining accurate information, especially among the elderly who may not immediately understand or respond to the questionnaire. Fifth, this study recruited the elderly from both urban and rural areas, and it will be useful to investigate geriatric health under different geographic and socioeconomic environments and lifestyles. However, this study also has certain limitations. No institutionalized elderly participants were included in this study; therefore some conclusions might not be directly applied to individuals living in long-term institutions. Furthermore, given that this is a cross-sectional study without follow-up, this may not assist in determining causal associations between predicted determinants of health and outcomes and may not be used to obtain trajectories of geriatric health over time.

The SABE will provide epidemiological information about determinants of active aging in Colombian elderly people. We expect our data to help find substantial body of evidence on how these determinants (and the interplay between them) have an influence during all life autonomy and independence. At the same time we expect identifying good predictors of health, for both individuals and populations, that permit the maintenance of autonomy and independence in aging. We insist on operationalizing active aging for epidemiological studies that in a near future could be compared among different cultures.

Two more features make SABE Colombia a highly valuable source for genuine cross-cultural comparisons. First, SABE Colombia is closely modeled and harmonized with its parent studies SABE in Latin America and Health Retirement Study in USA. This model has sparked and informed new research on aging over the world. Thus SABE Colombia is into a truly global perspective that permits comparisons with other epidemiological studies as SHARE (Europe), ELSA (England), or LASA (Amsterdam). Second, SABE Colombia is a nationwide survey including a large rural and urban sample. Thus cross-national comparisons will be made with other epidemiological studies in Colombia as SABE Bogotá and International Mobility in Aging Study (IMIAS).

The national survey system including SABE Colombia has projected system update thematic surveys every 10 years, so during that period this study will be the baseline for policy adjustment to aging. Through the Ministry of Health, the database will be released in the course of the first term of 2017 to be consulted not only by decision makers at the local, regional, and national level, but also by research groups interested in the elderly.

Comprehensive health data obtained from SABE Colombia based on active aging determinants is expected to contribute to policy formulation and planning of healthcare services, welfare management, and other social services for elderly persons in Colombia.

## Figures and Tables

**Table 1:** Operative categories of analysis based on active aging determinants in the SABE Colombia study.

Classification	Measures and instruments
Economic determinants	Work history: occupation, employment, current occupation, and reasons for no work. Income: subjective and objective financial status, household income, and expenditures. Social protection: pensions and social transfers. Marital status, ethnicity.
Social environment determinants	Educational attainment and degree of literacy. Assessment of frequency, size, and closeness of social support and social networks. Living arrangements and perception in respect to neighborhood safety. Early-life circumstances (first 15 years) and childhood adversity, abuse, mistreatment, and perceived discrimination. Social participation. Migration and displacement.
Physical environment determinants	Housing characteristics, housing safety, and environmental risks. Use of technology. Transportation services, built environment, and public services.
Personal determinants	Spirituality, sexuality, subjective health status, life space assessment, and functioning: physical and instrumental ADL limitations; mobility disability (Nagi questionnaire), grip strength, short physical performance battery (SPPB): balance, gait speed, and chair stands.
Behavioral determinants	Habits: smoking and alcohol. Physical activity: Advanced Activities of Daily Living scale. Mini-Nutritional Assessment. Anthropometry: height; weight; body mass index; circumference of waist, calf, and arm. Oral health.
Health and social services systems	Multimorbidity, chronic conditions (hypertension, osteoarthritis, diabetes, cardiovascular disease, osteoporosis, chronic obstructive pulmonary disease, cancer, and stroke), sensory impairments (vision and hearing loss), diseases linked to the aging processes (depression, dementia, falls and fear of falling, urinary incontinence, and frailty), mental well-being, and medications use (polypharmacy, hypnotics, homeopathic products, and adherence). Access and utilization of healthcare services.
Blood assays	Hemoglobin and hematocrit, total cholesterol, LDL, HDL cholesterol, and triglycerides.
Family caregiving	Caregiver characteristics, perceived burden, caregiver health, and training.
Qualitative data	Interactions and participation, physical and psychological well-being, experiences, relationships, social contacts and support, and living environment.

ADL: activities of daily living.

**Table 2:** Sociodemographic characteristics of participants at recruitment, 2015 (SABE Colombia).

	Residence				Sex					
	Urban		Rural		Men		Women		Total	
	%	95% CI	%	95% CI	%	95% CI	%	95% CI	%	95% CI
Age group										
60–64	77	61,5*–*87,6	23	12,4*–*38,5	47,1	44,4*–*49,7	52,9	50,3*–*55,6	30,6	28,8*–*32,5
65–69	79,3	63,9*–*89,3	20,7	10,7*–*36,1	46,6	43,4*–*49,8	53,4	50,2*–*56,6	26,6	24,6–28,7
70–74	76,3	60,9*–*86,9	23,7	13,1*–*39,1	45,5	43,1*–*47,9	54,5	52,1*–*56,9	16,8	16,0*–*17,7
75–79	80,6	66,9*–*89,5	19,4	10,5*–*33,1	43,5	38,9*–*48,1	56,5	51,9*–*61,1	13,4	12,7*–*14,2
80 and above	78,5	63,7*–*88,4	21,5	11,6*–*36,3	41,2	38,8*–*43,6	58,8	56,4*–*61,2	12,6	11,8*–*13,4
Skin color										
Light	84,5	70,0*–*92,7	15,5	7,3*–*30,0	41	39,1*–*42,8	59	57,2*–*60,9	54,2	46,2*–*61,9
Medium	72,3	55,7*–*84,4	27,7	15,6*–*44,3	49,8	46,1*–*53,4	50,2	46,6*–*53,9	34,7	30,3*–*39,5
Dark	65	47,2*–*79,5	35	20,5*–*52,8	54,2	50,2*–*58,2	45,8	41,8*–*49,8	11,1	7,6*–*15,9
Socioeconomic status										
1	55,6	41,4*–*68,9	44,4	31,1*–*58,6	46,8	44,5*–*49,3	53,2	50,7*–*55,5	28,4	19,5*–*39,4
2	78,8	61,6*–*89,5	21,2	10,5*–*38,4	45,1	43,2*–*47,0	54,9	53,0*–*56,8	39,6	36,5*–*42,8
3-4	97,2	89,8*–*99,3	2,8	0,7*–*10,2	45,7	43,7*–*47,8	54,3	52,2*–*56,3	30	22,1*–*39,4
5-6	99,7	97,7*–*100,0	0,3	0,0*–*2,3	28,7	16,7*–*44,8	71,3	55,2*–*83,3	2	1,3*–*3,1
Region										
Atlántico	75,4	47,6*–*91,2	24,6	8,8*–*52,4	46,1	42,4*–*49,9	53,9	50,1*–*57,6	19	8,0*–*38,9
Oriental	66,9	29,2*–*90,8	33,1	9,2*–*70,8	46	43,2*–*48,8	54	51,2*–*56,8	17,9	6,9*–*39,1
Central	77,5	34,9*–*95,7	22,5	4,3*–*65,1	45,3	42,4*–*48,1	54,7	51,9*–*57,6	27,1	9,4*–*57,4
Pacific	71	27,2*–*94,1	29	5,9*–*72,8	45,2	43,8*–*46,5	54,8	53,5*–*56,2	17,5	5,2*–*45,4
Orinoquia and Amazonia	96,3	83,3*–*99,3	3,7	0,7*–*16,7	47,8	43,7*–*51,8	52,2	48,2*–*56,3	1,4	0,4*–*4,4
Bogotá	99,8	96,7*–*100,0	0,2	0,0*–*3,3	44,5	44,5*–*44,5	55,5	55,5*–*55,5	17	2,5*–*62,1
Main cities										
Medellín	100		0		41,5	41,5*–*41,5	58,5	58,5*–*58,5	9,4	1,4*–*42,7
Cali	99,2	88,3*–*99,9	0,8	0,1*–*11,7	45,5	45,4*–*45,7	54,5	54,3*–*54,6	6,9	1,0*–*35,5
Barranquilla	100		0		36,6	36,6*–*36,6	63,4	63,4*–*63,4	3,6	0,5*–*22,7
Total	78,1	63,2*–*88,1	21,9	11,9*–*36,8	45,5	44,2*–*46,7	54,5	53,3*–*55,8	80,1	55,2*–*93,0
